# *cis*-Aqua­bis­(di-2-pyridyl­amine-κ^2^ *N*,*N*′)iodidomanganese(II) iodide

**DOI:** 10.1107/S1600536811047349

**Published:** 2011-11-12

**Authors:** Kwang Ha

**Affiliations:** a School of Applied Chemical Engineering, The Research Institute of Catalysis, Chonnam National University, Gwangju 500-757, Republic of Korea

## Abstract

The asymmetric unit of the title compound, [MnI(C_10_H_9_N_3_)_2_(H_2_O)]I, contains a cationic Mn^II^ complex and an I^−^ anion. In the complex, the Mn^II^ ion is six-coordinated in a considerably distorted *cis*-N_4_IO octa­hedral environment defined by four N atoms of the two chelating di-2-pyridyl­amine (dpa) ligands, one I^−^ anion and one O atom of a water ligand. As a result of the different *trans* effects of the I, N and O atoms, the Mn—N bond *trans* to the I atom is slightly longer than the Mn—N bond *trans* to the N or O atoms. The dpa ligands are not planar, with dihedral angles between the two pyridine rings of 26.2 (4) and 26.5 (4)°. The complex cations are stacked in columns along the *a* axis and are linked to the anions by inter­molecular O—H⋯I and N—H⋯I hydrogen bonds.

## Related literature

For the crystal structures of related Mn^II^ complexes with dpa, see: Bose *et al.* (2005 ▶).
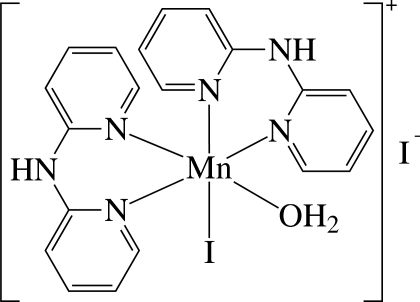


## Experimental

### 

#### Crystal data

[MnI(C_10_H_9_N_3_)_2_(H_2_O)]I
*M* *_r_* = 669.16
Triclinic, 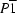

*a* = 8.598 (3) Å
*b* = 10.156 (3) Å
*c* = 13.909 (4) Å
α = 93.091 (6)°
β = 104.402 (6)°
γ = 98.262 (6)°
*V* = 1159.0 (6) Å^3^
*Z* = 2
Mo *K*α radiation
μ = 3.26 mm^−1^
*T* = 200 K
0.28 × 0.23 × 0.19 mm

#### Data collection

Bruker SMART 1000 CCD diffractometer
Absorption correction: multi-scan (*SADABS*; Bruker, 2000 ▶) *T*_min_ = 0.725, *T*_max_ = 1.000
7310 measured reflections
4509 independent reflections
3069 reflections with *I* > 2σ(*I*)
*R*_int_ = 0.041

#### Refinement

*R*[*F* ^2^ > 2σ(*F* ^2^)] = 0.050
*wR*(*F* ^2^) = 0.134
*S* = 1.06
4509 reflections
271 parameters
H-atom parameters constrained
Δρ_max_ = 1.01 e Å^−3^
Δρ_min_ = −1.13 e Å^−3^

### 

Data collection: *SMART* (Bruker, 2000 ▶); cell refinement: *SAINT* (Bruker, 2000 ▶); data reduction: *SAINT*; program(s) used to solve structure: *SHELXS97* (Sheldrick, 2008 ▶); program(s) used to refine structure: *SHELXL97* (Sheldrick, 2008 ▶); molecular graphics: *ORTEP-3* (Farrugia, 1997 ▶) and *PLATON* (Spek, 2009 ▶); software used to prepare material for publication: *SHELXL97*.

## Supplementary Material

Crystal structure: contains datablock(s) global, I. DOI: 10.1107/S1600536811047349/wm2558sup1.cif

Structure factors: contains datablock(s) I. DOI: 10.1107/S1600536811047349/wm2558Isup2.hkl

Additional supplementary materials:  crystallographic information; 3D view; checkCIF report

## Figures and Tables

**Table:** 

Mn1—O1	2.164 (6)
Mn1—N4	2.215 (6)
Mn1—N3	2.238 (6)
Mn1—N6	2.249 (6)
Mn1—N1	2.312 (6)
Mn1—I1	2.8785 (15)

**Table:** 

N4—Mn1—N6	81.0 (2)
N3—Mn1—N1	78.8 (2)

**Table 2:** Hydrogen-bond geometry (Å, °)

*D*—H⋯*A*	*D*—H	H⋯*A*	*D*⋯*A*	*D*—H⋯*A*
O1—H1*A*⋯I2^i^	0.84	2.76	3.500 (6)	148
O1—H1*B*⋯I1^ii^	0.84	2.73	3.490 (6)	152
N2—H2*N*⋯I2^iii^	0.92	2.77	3.681 (7)	172
N5—H5*N*⋯I2^iv^	0.92	2.80	3.710 (6)	173

Symmetry codes: (i) 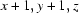; (ii) 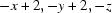; (iii) 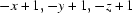; (iv) 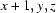.

**Fig. 1.**
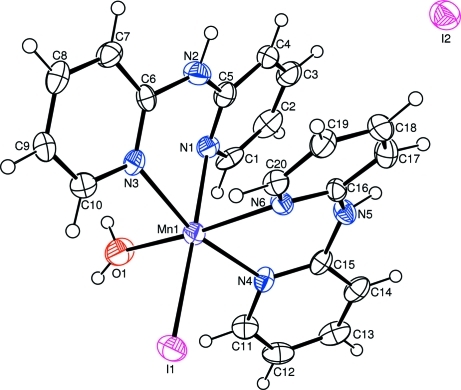
The molecular structure the title compound, with displacement ellipsoids drawn at the 40% probability level for non-H atoms.

**Fig. 2.**
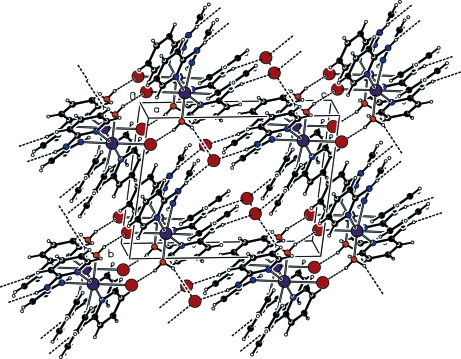
View of the unit-cell contents of the title compound. Hydrogen-bond interactions are drawn with dashed lines.

## supplementary crystallographic information

### Comment

Cationic Mn^II^ complexes with the di-2-pyridylamine (dpa; C_10_H_9_N_3_) ligand, such as [MnX(dpa)_2_(H_2_O)]ClO_4_ (*X* = N_3_^-^, NCO^-^), have been investigated previously (Bose *et al.*, 2005).

The asymmetric unit of the title compound, [MnI(dpa)_2_(H_2_O)]I, consists of a cationic Mn^II^ complex and an I^-^ anion (Fig. 1). In the complex, the Mn^II^ ion is six-coordinated in a considerably distorted *cis*-N_4_IO octahedral environment defined by four N atoms of the two chelating dpa ligands, one I^-^ anion and one O atom of a water ligand. The main contribution to the distortion is the tight N—Mn—N chelating angles (Table 1), which results in non-linear *trans* axes [N3—Mn1—N4 = 165.7 (2)° and O1—Mn1—N6 = 171.7 (2)°]. However, the apical I1—Mn1—N1 bond is almost linear with a bond angle of 177.61 (17)°. The Mn—N(dpa) bond lengths are somewhat different and longer than the Mn—O(H_2_O) bond (Table 1). As a result of the different *trans* effects of the I, N and O atoms, the Mn1—N1 bond *trans* to the I atom is slightly longer than the Mn—N bond *trans* to the N or O atoms. In the crystal structure, the dpa ligands are not planar. The dihedral angles between the two pyridyl rings of dpa are 26.2 (4)° and 26.5 (4)°.

The complexes are stacked in columns along the *a* axis, and the component cations and anions are linked by intermolecular O—H···I and N—H···I hydrogen bonds (Fig. 2, Table 2). In the column, numerous inter- and intramolecular π-π interactions between the pyridyl rings are present, the shortest centroid-centroid distance being 3.728 (5) Å.

### Experimental

To a solution of di-2-pyridylamine (0.3432 g, 2.005 mmol) in acetone (50 ml) was added MnI_2_ (0.3108 g, 1.007 mmol) and refluxed for 7 h. The formed precipitate was separated by filtration and washed with acetone, and dried at 323 K, to give a white powder (0.1571 g). Crystals suitable for X-ray analysis were obtained by slow evaporation from an MeOH solution.

### Refinement

Carbon-bound H atoms were positioned geometrically and allowed to ride on their respective parent atoms [C—H = 0.95 Å and *U*_iso_(H) = 1.2*U*_eq_(C)]. Nitrogen- and oxygen-bound H atoms were located from Fourier difference maps then allowed to ride on their parent atoms in the final cycles of refinement with N—H = 0.92 Å, O—H = 0.84 Å and *U*_iso_(H) = 1.5 *U*_eq_(N, O). The highest peak (1.01 e Å^-3^) and the deepest hole (-1.13 e Å^-3^) in the difference Fourier map are located 1.01 Å and 0.79 Å from the atoms C8 and I1, respectively.

### Crystal data

**Table:**

[MnI(C_10_H_9_N_3_)_2_(H_2_O)]I	*Z* = 2
*M**_r_* = 669.16	*F*(000) = 642
Triclinic, *P*1	*D*_x_ = 1.917 Mg m^−^^3^
Hall symbol: -P 1	Mo *K*α radiation, λ = 0.71073 Å
*a* = 8.598 (3) Å	Cell parameters from 2534 reflections
*b* = 10.156 (3) Å	θ = 2.5–25.7°
*c* = 13.909 (4) Å	µ = 3.26 mm^−^^1^
α = 93.091 (6)°	*T* = 200 K
β = 104.402 (6)°	Block, colorless
γ = 98.262 (6)°	0.28 × 0.23 × 0.19 mm
*V* = 1159.0 (6) Å^3^	

### Data collection

**Table:**

Bruker SMART 1000 CCD diffractometer	4509 independent reflections
Radiation source: fine-focus sealed tube	3069 reflections with *I* > 2σ(*I*)
graphite	*R*_int_ = 0.041
φ and ω scans	θ_max_ = 26.0°, θ_min_ = 2.0°
Absorption correction: multi-scan (*SADABS*; Bruker, 2000)	*h* = −9→10
*T*_min_ = 0.725, *T*_max_ = 1.000	*k* = −12→12
7310 measured reflections	*l* = −17→15

### Refinement

**Table:**

Refinement on *F*^2^	Primary atom site location: structure-invariant direct methods
Least-squares matrix: full	Secondary atom site location: difference Fourier map
*R*[*F*^2^ > 2σ(*F*^2^)] = 0.050	Hydrogen site location: inferred from neighbouring sites
*wR*(*F*^2^) = 0.134	H-atom parameters constrained
*S* = 1.06	*w* = 1/[σ^2^(*F*_o_^2^) + (0.0448*P*)^2^ + 2.3714*P*] where *P* = (*F*_o_^2^ + 2*F*_c_^2^)/3
4509 reflections	(Δ/σ)_max_ < 0.001
271 parameters	Δρ_max_ = 1.01 e Å^−^^3^
0 restraints	Δρ_min_ = −1.13 e Å^−^^3^

### Special details

**Table:**

Geometry. All e.s.d.'s (except the e.s.d. in the dihedral angle between two l.s. planes) are estimated using the full covariance matrix. The cell e.s.d.'s are taken into account individually in the estimation of e.s.d.'s in distances, angles and torsion angles; correlations between e.s.d.'s in cell parameters are only used when they are defined by crystal symmetry. An approximate (isotropic) treatment of cell e.s.d.'s is used for estimating e.s.d.'s involving l.s. planes.
Refinement. Refinement of *F*^2^ against ALL reflections. The weighted *R*-factor *wR* and goodness of fit *S* are based on *F*^2^, conventional *R*-factors *R* are based on *F*, with *F* set to zero for negative *F*^2^. The threshold expression of *F*^2^ > σ(*F*^2^) is used only for calculating *R*-factors(gt) *etc*. and is not relevant to the choice of reflections for refinement. *R*-factors based on *F*^2^ are statistically about twice as large as those based on *F*, and *R*- factors based on ALL data will be even larger.

### Fractional atomic coordinates and isotropic or equivalent isotropic displacement parameters (Å^2^)

**Table:**

	*x*	*y*	*z*	*U*_iso_*/*U*_eq_	
Mn1	0.92450 (15)	0.83226 (11)	0.17727 (9)	0.0334 (3)	
I1	0.70020 (7)	0.84510 (6)	−0.01259 (4)	0.0439 (2)	
O1	1.0782 (8)	1.0190 (6)	0.1727 (5)	0.0597 (19)	
H1A	1.1370	1.0566	0.2276	0.090*	
H1B	1.1097	1.0741	0.1359	0.090*	
N1	1.0972 (8)	0.8255 (6)	0.3329 (5)	0.0330 (15)	
N2	0.8918 (8)	0.8161 (7)	0.4170 (5)	0.0399 (17)	
H2N	0.8627	0.7822	0.4712	0.060*	
N3	0.7974 (8)	0.9327 (6)	0.2754 (5)	0.0362 (16)	
N4	1.0645 (8)	0.7020 (6)	0.1115 (5)	0.0320 (15)	
N5	1.0185 (8)	0.5213 (6)	0.2056 (5)	0.0348 (16)	
H5N	1.0725	0.4583	0.2387	0.052*	
N6	0.7948 (8)	0.6322 (6)	0.1996 (5)	0.0318 (15)	
C1	1.2452 (10)	0.7983 (8)	0.3321 (6)	0.040 (2)	
H1	1.2943	0.8345	0.2831	0.048*	
C2	1.3298 (11)	0.7210 (10)	0.3981 (7)	0.051 (3)	
H2	1.4363	0.7076	0.3966	0.061*	
C3	1.2553 (12)	0.6636 (9)	0.4664 (7)	0.048 (2)	
H3	1.3062	0.6038	0.5094	0.057*	
C4	1.1086 (11)	0.6936 (8)	0.4715 (6)	0.040 (2)	
H4	1.0567	0.6577	0.5194	0.048*	
C5	1.0354 (10)	0.7785 (8)	0.4046 (6)	0.0348 (19)	
C6	0.7953 (9)	0.9056 (7)	0.3694 (6)	0.0304 (18)	
C7	0.6994 (10)	0.9603 (8)	0.4199 (6)	0.0371 (19)	
H7	0.7061	0.9430	0.4872	0.045*	
C8	0.5937 (10)	1.0399 (8)	0.3740 (7)	0.040 (2)	
H8	0.5243	1.0762	0.4082	0.048*	
C9	0.5890 (11)	1.0668 (8)	0.2771 (6)	0.042 (2)	
H9	0.5148	1.1198	0.2424	0.051*	
C10	0.6939 (11)	1.0151 (8)	0.2330 (6)	0.042 (2)	
H10	0.6950	1.0382	0.1679	0.050*	
C11	1.1421 (10)	0.7549 (9)	0.0432 (6)	0.038 (2)	
H11	1.1232	0.8405	0.0232	0.046*	
C12	1.2430 (10)	0.6932 (10)	0.0028 (6)	0.046 (2)	
H12	1.2942	0.7343	−0.0436	0.055*	
C13	1.2698 (10)	0.5664 (10)	0.0317 (6)	0.046 (2)	
H13	1.3415	0.5209	0.0055	0.055*	
C14	1.1937 (10)	0.5088 (9)	0.0966 (6)	0.041 (2)	
H14	1.2095	0.4222	0.1155	0.050*	
C15	1.0906 (9)	0.5798 (7)	0.1355 (6)	0.0314 (18)	
C16	0.8692 (9)	0.5266 (7)	0.2229 (6)	0.0303 (18)	
C17	0.7977 (11)	0.4190 (8)	0.2649 (6)	0.042 (2)	
H17	0.8529	0.3454	0.2814	0.050*	
C18	0.6502 (12)	0.4210 (8)	0.2814 (6)	0.046 (2)	
H18	0.6030	0.3504	0.3125	0.055*	
C19	0.5665 (10)	0.5270 (8)	0.2529 (6)	0.038 (2)	
H19	0.4609	0.5288	0.2620	0.046*	
C20	0.6419 (9)	0.6271 (8)	0.2117 (6)	0.0312 (18)	
H20	0.5845	0.6979	0.1900	0.037*	
I2	0.23491 (7)	0.28327 (5)	0.35974 (4)	0.03842 (18)	

### Atomic displacement parameters (Å^2^)

**Table:**

	*U*^11^	*U*^22^	*U*^33^	*U*^12^	*U*^13^	*U*^23^
Mn1	0.0398 (7)	0.0302 (6)	0.0334 (7)	0.0067 (6)	0.0144 (6)	0.0048 (5)
I1	0.0482 (4)	0.0497 (4)	0.0375 (3)	0.0134 (3)	0.0131 (3)	0.0129 (3)
O1	0.084 (5)	0.040 (3)	0.054 (4)	−0.021 (3)	0.034 (4)	−0.003 (3)
N1	0.034 (4)	0.031 (4)	0.033 (4)	−0.001 (3)	0.012 (3)	0.000 (3)
N2	0.043 (4)	0.047 (4)	0.036 (4)	0.015 (3)	0.016 (3)	0.011 (3)
N3	0.045 (4)	0.031 (4)	0.040 (4)	0.017 (3)	0.018 (3)	0.002 (3)
N4	0.030 (4)	0.026 (3)	0.042 (4)	0.003 (3)	0.014 (3)	−0.001 (3)
N5	0.034 (4)	0.033 (4)	0.042 (4)	0.013 (3)	0.013 (3)	0.011 (3)
N6	0.035 (4)	0.028 (3)	0.029 (4)	0.002 (3)	0.005 (3)	0.005 (3)
C1	0.031 (5)	0.051 (5)	0.032 (5)	−0.003 (4)	0.008 (4)	−0.017 (4)
C2	0.032 (5)	0.064 (6)	0.051 (6)	0.013 (5)	0.003 (4)	−0.014 (5)
C3	0.057 (6)	0.048 (5)	0.037 (5)	0.021 (5)	0.002 (5)	−0.003 (4)
C4	0.054 (6)	0.030 (4)	0.033 (5)	0.002 (4)	0.009 (4)	0.001 (4)
C5	0.032 (5)	0.031 (4)	0.037 (5)	0.003 (4)	0.004 (4)	−0.004 (4)
C6	0.031 (4)	0.019 (4)	0.040 (5)	−0.002 (3)	0.009 (4)	0.005 (3)
C7	0.039 (5)	0.036 (5)	0.039 (5)	0.005 (4)	0.018 (4)	−0.002 (4)
C8	0.037 (5)	0.031 (4)	0.055 (6)	0.010 (4)	0.015 (4)	−0.005 (4)
C9	0.056 (6)	0.030 (4)	0.043 (5)	0.018 (4)	0.011 (4)	0.008 (4)
C10	0.053 (6)	0.039 (5)	0.038 (5)	0.011 (4)	0.019 (4)	0.005 (4)
C11	0.031 (5)	0.047 (5)	0.041 (5)	0.006 (4)	0.015 (4)	0.005 (4)
C12	0.036 (5)	0.070 (6)	0.034 (5)	0.000 (5)	0.015 (4)	0.005 (5)
C13	0.027 (5)	0.073 (7)	0.040 (5)	0.016 (4)	0.011 (4)	−0.008 (5)
C14	0.033 (5)	0.047 (5)	0.039 (5)	0.011 (4)	−0.001 (4)	−0.011 (4)
C15	0.019 (4)	0.031 (4)	0.036 (4)	−0.003 (3)	0.000 (3)	−0.011 (4)
C16	0.035 (4)	0.023 (4)	0.029 (4)	0.001 (3)	0.002 (3)	0.002 (3)
C17	0.042 (5)	0.032 (4)	0.051 (5)	0.013 (4)	0.007 (4)	0.005 (4)
C18	0.062 (6)	0.034 (5)	0.040 (5)	−0.006 (4)	0.017 (5)	0.006 (4)
C19	0.038 (5)	0.035 (5)	0.042 (5)	0.004 (4)	0.014 (4)	−0.002 (4)
C20	0.025 (4)	0.038 (4)	0.034 (4)	0.014 (4)	0.007 (3)	0.003 (4)
I2	0.0426 (3)	0.0392 (3)	0.0373 (3)	0.0124 (3)	0.0130 (2)	0.0094 (2)

### Geometric parameters (Å, °)

**Table:**

Mn1—O1	2.164 (6)	C3—H3	0.9500
Mn1—N4	2.215 (6)	C4—C5	1.399 (11)
Mn1—N3	2.238 (6)	C4—H4	0.9500
Mn1—N6	2.249 (6)	C6—C7	1.364 (10)
Mn1—N1	2.312 (6)	C7—C8	1.366 (11)
Mn1—I1	2.8785 (15)	C7—H7	0.9500
O1—H1A	0.8400	C8—C9	1.383 (12)
O1—H1B	0.8400	C8—H8	0.9500
N1—C5	1.321 (10)	C9—C10	1.356 (11)
N1—C1	1.343 (9)	C9—H9	0.9500
N2—C5	1.391 (10)	C10—H10	0.9500
N2—C6	1.401 (9)	C11—C12	1.349 (11)
N2—H2N	0.9200	C11—H11	0.9500
N3—C6	1.355 (10)	C12—C13	1.403 (13)
N3—C10	1.359 (10)	C12—H12	0.9500
N4—C15	1.338 (10)	C13—C14	1.351 (12)
N4—C11	1.381 (10)	C13—H13	0.9500
N5—C16	1.371 (10)	C14—C15	1.407 (11)
N5—C15	1.395 (10)	C14—H14	0.9500
N5—H5N	0.9200	C16—C17	1.403 (11)
N6—C16	1.339 (9)	C17—C18	1.347 (12)
N6—C20	1.361 (9)	C17—H17	0.9500
C1—C2	1.378 (12)	C18—C19	1.401 (11)
C1—H1	0.9500	C18—H18	0.9500
C2—C3	1.382 (13)	C19—C20	1.356 (11)
C2—H2	0.9500	C19—H19	0.9500
C3—C4	1.358 (12)	C20—H20	0.9500
			
O1—Mn1—N4	96.3 (2)	N1—C5—N2	119.4 (7)
O1—Mn1—N3	91.1 (2)	N1—C5—C4	123.5 (8)
N4—Mn1—N3	165.7 (2)	N2—C5—C4	117.1 (8)
O1—Mn1—N6	171.7 (2)	N3—C6—C7	122.5 (7)
N4—Mn1—N6	81.0 (2)	N3—C6—N2	119.7 (7)
N3—Mn1—N6	89.9 (2)	C7—C6—N2	117.8 (7)
O1—Mn1—N1	85.5 (2)	C6—C7—C8	120.1 (8)
N4—Mn1—N1	89.6 (2)	C6—C7—H7	120.0
N3—Mn1—N1	78.8 (2)	C8—C7—H7	120.0
N6—Mn1—N1	86.7 (2)	C7—C8—C9	119.0 (8)
O1—Mn1—I1	94.82 (18)	C7—C8—H8	120.5
N4—Mn1—I1	92.68 (17)	C9—C8—H8	120.5
N3—Mn1—I1	98.81 (18)	C10—C9—C8	117.8 (8)
N6—Mn1—I1	93.11 (16)	C10—C9—H9	121.1
N1—Mn1—I1	177.61 (17)	C8—C9—H9	121.1
Mn1—O1—H1A	116.6	C9—C10—N3	124.7 (8)
Mn1—O1—H1B	145.6	C9—C10—H10	117.6
H1A—O1—H1B	97.7	N3—C10—H10	117.6
C5—N1—C1	116.5 (7)	C12—C11—N4	124.4 (8)
C5—N1—Mn1	118.9 (5)	C12—C11—H11	117.8
C1—N1—Mn1	114.9 (5)	N4—C11—H11	117.8
C5—N2—C6	131.9 (7)	C11—C12—C13	117.8 (8)
C5—N2—H2N	112.5	C11—C12—H12	121.1
C6—N2—H2N	115.2	C13—C12—H12	121.1
C6—N3—C10	115.7 (7)	C14—C13—C12	120.1 (8)
C6—N3—Mn1	126.3 (5)	C14—C13—H13	119.9
C10—N3—Mn1	117.4 (5)	C12—C13—H13	119.9
C15—N4—C11	115.9 (7)	C13—C14—C15	118.6 (8)
C15—N4—Mn1	126.9 (5)	C13—C14—H14	120.7
C11—N4—Mn1	117.1 (5)	C15—C14—H14	120.7
C16—N5—C15	130.6 (6)	N4—C15—N5	119.0 (7)
C16—N5—H5N	114.2	N4—C15—C14	123.1 (8)
C15—N5—H5N	114.4	N5—C15—C14	117.8 (7)
C16—N6—C20	117.4 (7)	N6—C16—N5	120.3 (7)
C16—N6—Mn1	123.8 (5)	N6—C16—C17	121.6 (8)
C20—N6—Mn1	117.2 (5)	N5—C16—C17	118.2 (7)
N1—C1—C2	123.8 (8)	C18—C17—C16	119.3 (8)
N1—C1—H1	118.1	C18—C17—H17	120.3
C2—C1—H1	118.1	C16—C17—H17	120.3
C1—C2—C3	118.1 (9)	C17—C18—C19	120.1 (8)
C1—C2—H2	121.0	C17—C18—H18	120.0
C3—C2—H2	121.0	C19—C18—H18	120.0
C4—C3—C2	119.2 (8)	C20—C19—C18	117.5 (8)
C4—C3—H3	120.4	C20—C19—H19	121.3
C2—C3—H3	120.4	C18—C19—H19	121.3
C3—C4—C5	118.5 (8)	C19—C20—N6	124.0 (7)
C3—C4—H4	120.8	C19—C20—H20	118.0
C5—C4—H4	120.8	N6—C20—H20	118.0
			
O1—Mn1—N1—C5	142.6 (6)	Mn1—N1—C5—C4	138.1 (6)
N4—Mn1—N1—C5	−121.0 (6)	C6—N2—C5—N1	−4.3 (12)
N3—Mn1—N1—C5	50.6 (6)	C6—N2—C5—C4	174.6 (7)
N6—Mn1—N1—C5	−40.0 (6)	C3—C4—C5—N1	3.9 (12)
O1—Mn1—N1—C1	−72.4 (6)	C3—C4—C5—N2	−174.9 (7)
N4—Mn1—N1—C1	23.9 (6)	C10—N3—C6—C7	−1.9 (11)
N3—Mn1—N1—C1	−164.5 (6)	Mn1—N3—C6—C7	−172.1 (6)
N6—Mn1—N1—C1	105.0 (6)	C10—N3—C6—N2	176.7 (7)
O1—Mn1—N3—C6	−117.8 (6)	Mn1—N3—C6—N2	6.5 (10)
N4—Mn1—N3—C6	3.7 (14)	C5—N2—C6—N3	26.0 (12)
N6—Mn1—N3—C6	54.0 (6)	C5—N2—C6—C7	−155.4 (8)
N1—Mn1—N3—C6	−32.6 (6)	N3—C6—C7—C8	3.7 (12)
I1—Mn1—N3—C6	147.1 (6)	N2—C6—C7—C8	−174.9 (7)
O1—Mn1—N3—C10	72.1 (6)	C6—C7—C8—C9	−1.8 (12)
N4—Mn1—N3—C10	−166.3 (8)	C7—C8—C9—C10	−1.7 (12)
N6—Mn1—N3—C10	−116.1 (6)	C8—C9—C10—N3	3.7 (13)
N1—Mn1—N3—C10	157.3 (6)	C6—N3—C10—C9	−1.9 (12)
I1—Mn1—N3—C10	−22.9 (6)	Mn1—N3—C10—C9	169.2 (7)
O1—Mn1—N4—C15	143.8 (6)	C15—N4—C11—C12	−1.9 (12)
N3—Mn1—N4—C15	22.8 (14)	Mn1—N4—C11—C12	174.1 (7)
N6—Mn1—N4—C15	−28.3 (6)	N4—C11—C12—C13	0.6 (13)
N1—Mn1—N4—C15	58.4 (6)	C11—C12—C13—C14	1.1 (13)
I1—Mn1—N4—C15	−121.0 (6)	C12—C13—C14—C15	−1.2 (12)
O1—Mn1—N4—C11	−31.7 (6)	C11—N4—C15—N5	178.8 (7)
N3—Mn1—N4—C11	−152.7 (9)	Mn1—N4—C15—N5	3.2 (10)
N6—Mn1—N4—C11	156.2 (6)	C11—N4—C15—C14	1.6 (11)
N1—Mn1—N4—C11	−117.1 (6)	Mn1—N4—C15—C14	−173.9 (5)
I1—Mn1—N4—C11	63.4 (5)	C16—N5—C15—N4	36.6 (11)
N4—Mn1—N6—C16	36.5 (6)	C16—N5—C15—C14	−146.2 (8)
N3—Mn1—N6—C16	−132.5 (6)	C13—C14—C15—N4	−0.2 (12)
N1—Mn1—N6—C16	−53.7 (6)	C13—C14—C15—N5	−177.3 (7)
I1—Mn1—N6—C16	128.7 (6)	C20—N6—C16—N5	175.3 (7)
N4—Mn1—N6—C20	−158.5 (6)	Mn1—N6—C16—N5	−19.7 (9)
N3—Mn1—N6—C20	32.5 (5)	C20—N6—C16—C17	−4.4 (10)
N1—Mn1—N6—C20	111.3 (5)	Mn1—N6—C16—C17	160.5 (6)
I1—Mn1—N6—C20	−66.3 (5)	C15—N5—C16—N6	−27.0 (12)
C5—N1—C1—C2	2.9 (11)	C15—N5—C16—C17	152.7 (8)
Mn1—N1—C1—C2	−142.9 (7)	N6—C16—C17—C18	0.6 (12)
N1—C1—C2—C3	2.8 (12)	N5—C16—C17—C18	−179.2 (8)
C1—C2—C3—C4	−5.2 (13)	C16—C17—C18—C19	2.7 (12)
C2—C3—C4—C5	2.1 (12)	C17—C18—C19—C20	−2.0 (12)
C1—N1—C5—N2	172.5 (6)	C18—C19—C20—N6	−2.1 (12)
Mn1—N1—C5—N2	−43.2 (9)	C16—N6—C20—C19	5.3 (11)
C1—N1—C5—C4	−6.3 (11)	Mn1—N6—C20—C19	−160.7 (6)

### Hydrogen-bond geometry (Å, °)

**Table:**

*D*—H···*A*	*D*—H	H···*A*	*D*···*A*	*D*—H···*A*
O1—H1A···I2^i^	0.84	2.76	3.500 (6)	148.
O1—H1B···I1^ii^	0.84	2.73	3.490 (6)	152.
N2—H2N···I2^iii^	0.92	2.77	3.681 (7)	172.
N5—H5N···I2^iv^	0.92	2.80	3.710 (6)	173.

Symmetry codes: (i) *x*+1, *y*+1, *z*; (ii) −*x*+2, −*y*+2, −*z*; (iii) −*x*+1, −*y*+1, −*z*+1; (iv) *x*+1, *y*, *z*.
